# Post-traumatic Ruptured Scrotal Collection: Pyocele or Hematocele?

**DOI:** 10.7759/cureus.45198

**Published:** 2023-09-13

**Authors:** Anshul Sood, Gaurav V Mishra, Shreya Khandelwal, Keyur Saboo, Manasa Suryadevara

**Affiliations:** 1 Radiodiagnosis, Datta Meghe Institute of Higher Education and Research, Jawaharlal Nehru Medical College, Wardha, IND; 2 Internal Medicine, Datta Meghe Institute of Higher Education and Research, Jawaharlal Nehru Medical College, Wardha, IND

**Keywords:** pyocele, testicular rupture, usg, ultrasound, falling snow

## Abstract

Ultrasound is the modality of choice for detecting the causes of acute scrotal pain and diagnosing scrotal pathologies. Pyocele is a term used for describing the purulent fluid collection in the scrotal sac, which may present to the hospital as a complication of testicular abscess, epididymal-orchitis, or post-trauma. Ultrasound is the modality of choice for diagnosing such pathologies. The presented case is of a young male from rural India who developed a pyocele post-trauma and underwent an ultrasound examination, where it was found to be a ruptured pyocele with contents spreading into the hemi-scrotum. Rupture of the tunica is frequently associated with the hematocele; however, pyocele is less commonly associated with rupture. The case report discusses the causes of pyocele, imaging features, management, and complications of this pathology along with other possible differential diagnosis.

## Introduction

Scrotal pyocele, a rare urological emergency, is defined as a purulent collection between the visceral and parietal tunica vaginalis layers surrounding the testicle. Pyocele may develop through sexually transmitted infections, intra-abdominal infections, or due to trauma [1-3]. Ultrasound is the imaging modality of choice for diagnosing scrotal pathologies [4]. Patients are initially treated with fluid resuscitation, empirical antibiotics, and pain management, but in a few cases, empirical treatments are useless, and emergency surgical drainage is necessary [5].

## Case presentation

A 28-year-old male came to the emergency department with complaints of pain in the left hemi-scrotum which was appearing red and swollen with a gradual increase in the swelling for three days. On inspection, the left hemi-scrotum appears red and swollen with loss of crease, giving a smooth and shiny appearance. On examination, the scrotum was hot and tender on touch with signs of inflammation. The patient was advised to undergo an ultrasound investigation to determine the cause of the pain and swelling.

**Figure 1:**
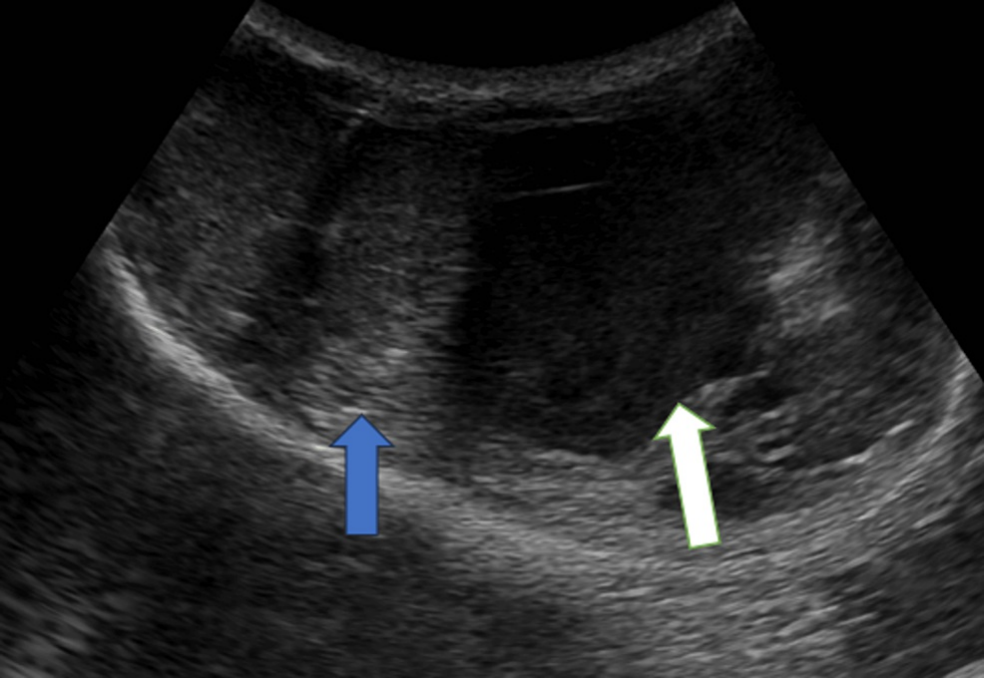
Ultrasound image of the scrotal sac using curvi-linear probe showing hypoechoic collection in the left scrotal sac (white arrow) with a displacement of the left testes away from the midline toward the right (blue arrow).

On ultrasound investigation, there was a heterogeneous collection between the scrotal sac and testes with multiple hyper-echogenic debris within, giving a falling snow sign. The collection measured approximately 6 x 5 x 3 cm and was causing a mass effect in the form of displacement of the left testes towards the right as shown in Figure 1.

**Figure 2:**
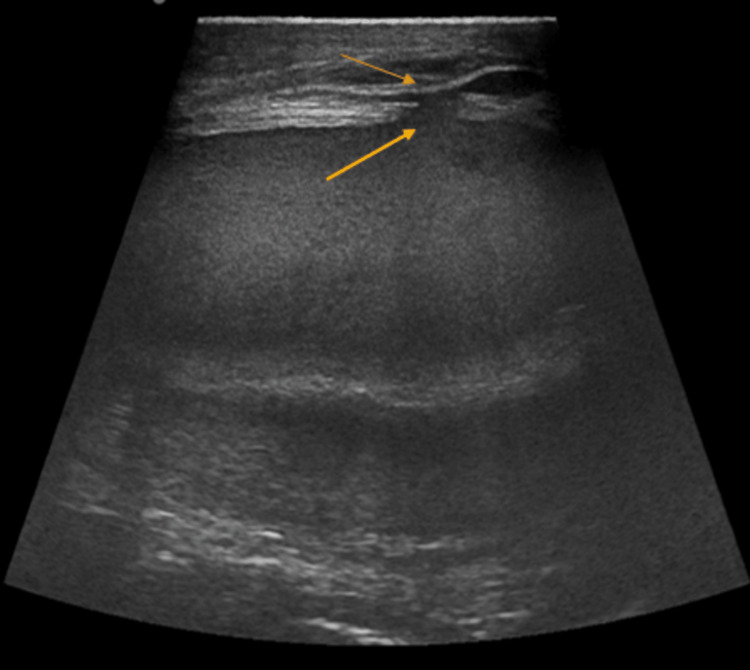
Ultrasound image of the left scrotal sac using linear probe showing hypoechoic collection with debris giving falling snow appearance with disruption in the tunica layers (yellow arrow), and intact tunica vaginalis layer (orange arrow).

The collection was noted adjacent to the left testicle, and there was a disruption in the continuity of the tunica layers with defect of size approximately 1 cm. The contents are noted to pass out of the ruptured membrane and visceral tunica vaginalis appears intact, as shown in Figure 2.

No calcification or air foci were noted within the collection. The size, shape, vascularity, and echotexture of the right testicle appear normal. The patient has a history of trauma to the scrotal sac seven days ago when a rod hit the scrotal sac while working in a field. No significant history of unprotected sexual intercourse, urinary tract infection or sexually transmitted infection.

The patient underwent emergency surgical exploration with debridement and excision of the devitalized tissues. A tissue culture was sent to the microbiology department to find out the pathogen behind the formation of the pyocele. The patient was then discharged and advised to follow up after three weeks to check for the status of the post-operative scrotum.

## Discussion

The purulent collection between the visceral and parietal tunica vaginalis layers surrounding the testes is called pyocele. There are various routes through which micro-organisms reach the scrotal sac, which may include transmission via the sexual route, intra-abdominal infections, or trauma [1-3].

Epididymo-orchitis and epididymitis are the most common causes of acute presentation of scrotal pain, which, when left untreated, might lead to an abscess formation or infarction [6]. Neisseria gonorrhoeae and Chlamydia trachomatis are the two most common pathogens in sexually active males younger than 35 years of age. In men older than 35 years, pathogens contaminating the urinary tract, such as E. coli, are the common pathogens [6].

Hydrocele is an acquired or congenital serous fluid collection between the two layers of tunica vaginalis and is the most common cause of painless scrotal enlargement. Ultrasound shows simple fluid collection, which shows no vascularity on the color Doppler. Sometimes, the collection may be septated and may occasionally contain calcifications and cholesterol [7].

Hematocele is a vital differential that develops as a sequel to trauma to the scrotum, occasionally after surgical intervention. Some authors report that varicocele is considered a significant risk factor for the development of hematocele. Ultrasound is the first-line imaging modality which shows collection with increased echogenicity and often septa within. In cases of fresh hematocele, the collection might appear anechoic, though some turbulence will occasionally be seen within it [8]. The presence of air foci within the collection helps in differentiating pyocele and hematocele [9].

Testicular trauma is the third most common cause of acute scrotal pain [10]. Various causes for acute scrotal pain include testicular fracture, dislocation, torsion, intratesticular pseudoaneurysm, or intratesticular hematoma. In cases of testicular rupture, the signs of disruption of the tunica albuginea, like loss of continuity, crinkling, or retraction, may be seen. Also, sometimes extrusion of the seminiferous tubules might occur, which can be assessed using color Doppler. Color Doppler will retain blood supply and can be differentiated from a complex hematocele [10].

Pyocele can be managed conservatively by fluid resuscitation, pain management, and empirical antibiotics [5]. However, immediate surgical exploration, debridement, and removal of the devitalized testicular tissue with the closure of the ruptured membranes form the mainstay of the treatment [11].

The presented case had a history of trauma, following which the patient developed pyocele. He underwent an ultrasound investigation, which confirmed the presence of heterogenous collection between the two layers of tunica vaginalis with rupture of the tunica and leakage of contents outside. Immediate surgical exploration with debridement and excision of the devitalized tissues was performed.

Complications may include Fournier gangrene, a necrotizing infection involving both the superficial and deep facial planes and can be fatal if not treated in time. Computed tomography is the modality of choice for diagnosing Fournier gangrene [6].

## Conclusions

Pyocele is an acute scrotal emergency that needs immediate management. Hematocele must be considered the first differential diagnosis in cases of ruptured membranes of the scrotal sac; however, pyocele should not be simply ruled out. Early detection of the cause of acute scrotal pain must be done with prompt, timely management to prevent any complications. Surgical management remains the gold standard treatment for the pyocele.
